# Brain parenchyma deformation and strain in patients with spontaneous intracranial hypotension

**DOI:** 10.1186/s12987-026-00865-8

**Published:** 2026-08-27

**Authors:** Amir El Rahal, Jürgen Beck, Katharina Wolf, Florian Volz, Manou Overstijns, Niklas Lützen, Charlotte Zander, Horst Urbach, Pierre Scheffler

**Affiliations:** 1 Department of Neurosurgery, Medical Center–University of Freiburg, Breisacher-Str. 64, 79106 Freiburg im Breisgau, Germany; 2 German CSF Center, Medical Center–University of Freiburg, Freiburg, Germany; 3 Department of Diagnostic and Interventional Neuroradiology, Medical Center–University of Freiburg, Freiburg, Germany; 4 Department of Diagnostic and Interventional Radiology, Medical Center–University of Freiburg, Freiburg, Germany

**Keywords:** Spontaneous intracranial hypotension, SIH, Brain sagging, Brain shift, AI, Convolutional neural network, Surgical closure, CSF Leak, CSF-venous fistula, Neurosurgery

## Abstract

**Background:**

Spontaneous intracranial hypotension (SIH) is mainly associated with orthostatic headache but can also cause cognitive impairment, with symptoms mimicking behavioral-type frontotemporal dementia in severe cases. SIH is caused by a spinal cerebrospinal fluid (CSF) leak and can lead to brain sagging. The specific brain regions affected by this motion, their deformation, and the magnitude of the associated mechanical strain have not been quantitatively characterized. We investigated whether brain MRI in patients with SIH shows quantifiable strain, expansion, and compression of brain structures by comparing images before and after surgical occlusion of a spinal leak.

**Methods:**

We performed a retrospective analysis of 96 patients with SIH who underwent surgical closure of a spinal CSF leak. We performed AI-assisted diffeomorphic registration of pre- and postoperative MRI. From the resulting displacement maps, we calculated the Jacobian determinant as an indicator of expansion and contraction, and the von Mises equivalent deviatoric strain as an indicator of distortion and shearing in brain regions. Results were compared with 61 healthy adults scanned twice over a comparable interval, and correlated with perioperative headache, quality-of-life and cognitive scores.

**Results:**

Ventricles and transverse sinuses showed relevant perioperative expansion and contraction, respectively. Beyond these expected volumetric changes, significant deviatoric strain was observed in temporomesial and frontal structures, including the temporal poles (right/ left: 8.6/ 8.7%), hippocampus (9.0/ 9.8%), and basal forebrain (9.9/ 9.8%) (*p* < 0.001 for all), despite only moderate volumetric change in these regions. Deformation substantially exceeded that of healthy controls. Whole-brain strain showed a modest positive association with perioperative improvement in headache burden, whereas analyses of cognitive outcome were underpowered.

**Conclusion:**

SIH is associated with quantifiable regional brain deformation resulting in region-specific mechanical strain. Elevated strain in fronto-limbic and temporomesial regions, implicated in behavioral and cognitive regulation, suggests a network-level mechanical effect that may contribute to the clinical presentation observed in severe SIH.

**Supplementary Information:**

The online version contains supplementary material available at https://doi.org/10.1186/s12987-026-00865-8.

## Background

Spontaneous intracranial hypotension (SIH) is an underdiagnosed condition caused by a cerebrospinal fluid (CSF) leak along the spine [1]. While orthostatic headache is the hallmark symptom, SIH can produce a wide spectrum of manifestations, including tinnitus, diplopia, and cognitive or behavioral changes. In severe and prolonged cases, patients may develop neuropsychiatric symptoms that closely mimic the behavioral variant of frontotemporal dementia (bvFTD) [2–5]. This overlap is increasingly recognized and has given rise to the concept of “spinal dementia” [6–8].

Magnetic resonance imaging (MRI) is central to the diagnosis and follow-up of SIH. Spinal CSF leaks are associated with characteristic qualitative brain MRI findings, commonly termed “brain sagging” [9]. Some of these imaging features have been aggregated into an SIH-specific scoring system, including reduced CSF cistern size, venous engorgement, pachymeningeal enhancement, and subdural effusions [10]. However, these changes remain mainly descriptive, and importantly, brain sagging represents not only simple, uniform downward displacement of intracranial structures as a whole, but also deformation of the brain parenchyma and surrounding structures. Such deformation may include both volumetric redistribution and distortional strain, reflecting mechanical stress that may vary between anatomical regions. To date, it remains unknown which specific regions bear the greatest mechanical burden and whether that burden is distributed uniformly or concentrates in functionally eloquent networks. Advances in artificial intelligence now enable automated, reproducible quantitative analysis of structural MRI, allowing for objective, voxel-wise measurement of brain deformation. A quantitative assessment of strain across functionally connected brain areas could help explain the diversity of clinical manifestations in SIH, including the cognitive and behavioral changes related to CSF leaks, and enable development of quantitative deformation biomarkers.

In this study, we aimed to investigate and characterize regional patterns of mechanical deformation and strain in specific brain regions in SIH patients before and after surgical closure of a spinal leak.

## Methods

### Ethics approval

Our study protocol was approved by the local ethics committee (IDs: 22-1512-S1-retro/ 24-1296-S1-retro); the study was conducted in accordance with good clinical practice.

### Participants

For this single-center study from a tertiary referral center, we used a local MRI dataset of patients who met the ICHD-3 criteria for SIH and underwent surgery for a spinal CSF leak between April 2018 and December 2024 at our center, and who had adequate pre- and postoperative MRI imaging [11]. Patients were required to have pre- and postoperative brain MRI scans with isotropic contrast-enhanced T1-weighted sequences at least 1 mm in resolution. Preoperative imaging had to be acquired within 12 months prior to surgery; postoperative imaging was required within 1–12 months after surgery, preferably at the 3-month follow-up. If multiple scans were available, the most recent preoperative and the postoperative scan closest to follow-up were selected. MRI images were also screened to exclude those showing subdural effusions or significant metal artifacts.

As a control group, we used the Beijing Normal University 2 (BNU 2) dataset, a publicly available dataset published by the Consortium for Reliability and Reproducibility (CoRR) [12]. The dataset includes 61 healthy adults between 19 and 23 years old who underwent two T1 MRI scans at least three months apart (103–189 days).

### Image processing

**Fig. 1:**
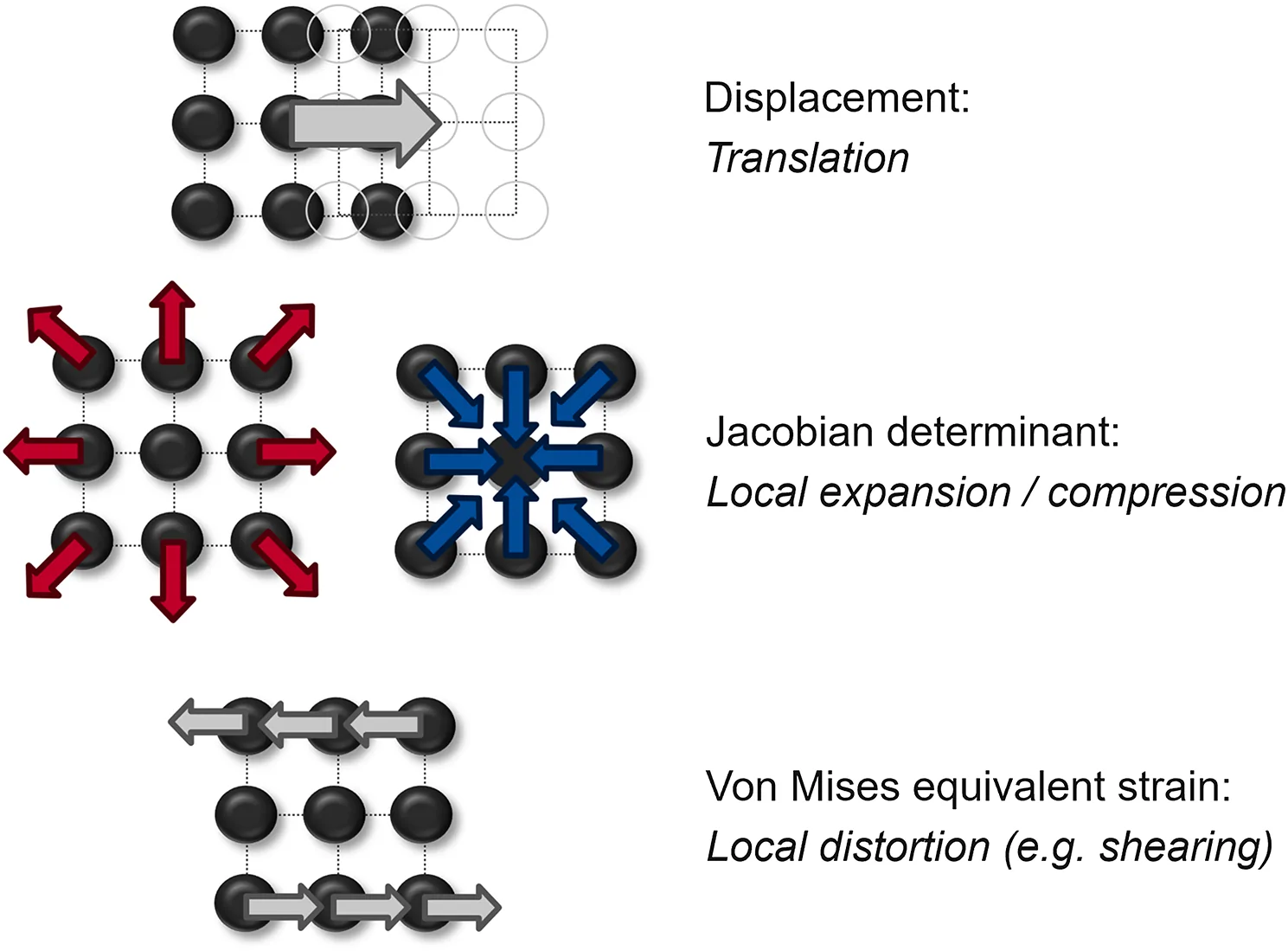
Illustration showing interpretations of the values generated for the study; the black spheres represent individual voxels within a grid. The displacement map is generated through diffeomorphic registration of pre- and postoperative images and shows voxel-wise translation movements in three-dimensional space. The displacement map is used to calculate the Jacobian determinant, for which values below 1 indicate local compression and values above 1 indicate local expansion. The von Mises equivalent strain indicates any deviatoric strain irrespective of expansion or compression

Image processing was automated with Python scripts. MRI data were converted from DICOM to NIfTI format. Automated segmentation of 155 brain regions and CSF spaces, as described by the Desikan-Killiany-Tourville protocol, was performed using *AssemblyNet*, followed by visual verification of all segmentations by two authors (PS, AER) [13, 14]. Diffeomorphic registration between pre- and postoperative scans was performed with *SynthMorph* to generate a voxel-wise displacement field $\:\left(\mathbf{u}\right)$ [15]. The displacement field was then used to calculate the Jacobian determinant and the von Mises equivalent strain; the interpretation of these values is illustrated in Fig. 1.

### Quantification of expansion and compression

Local changes in tissue volume were quantified using the Jacobian determinant (* J*), a standard measure of local volume change in tensor-based morphometry [16, 17]. We first defined the deformation gradient tensor as $\:\mathbf{F}=\mathbf{I}+\:\nabla\:\mathbf{u}$ where $\:\mathbf{I}$ is the identity matrix and $\:\nabla\:\mathbf{u}$ is the gradient of the displacement field. The determinant of this matrix was calculated at each voxel:

$$\:J\:=\mathrm{d}\mathrm{e}\mathrm{t}\left(\mathbf{F}\right)$$

A value of * J>1* indicates local volumetric expansion, while * J* < 1 indicates local compression. For example, * J=1.1* would indicate a 10% local volume expansion.

### Quantification of deviatoric strain

The Jacobian determinant captures changes in volume but not changes in shape. To quantify the shape-distorting deformation, we first calculated the Green-Lagrange strain tensor $\:\mathbf{E}$. This measure ensures rotational invariance, meaning that global head tilting does not introduce artificial strain values:

$$\:\mathbf{E}=\frac{1}{2}({\mathbf{F}}^{\mathrm{T}}\mathbf{F}-\mathbf{I})$$

At each voxel, $\:\mathbf{E}\:$ is a symmetric 3 × 3 tensor that carries the volumetric component in the sum of its diagonal (the trace, denoted $\:\mathrm{t}\mathrm{r}$). We then isolate shape change by taking the deviatoric strain $\:\mathrm{e}$:

$$\:\mathrm{e}=\mathbf{E}-\frac{1}{3}\mathrm{t}\mathrm{r}\left(\mathbf{E}\right)\mathbf{I}$$

Consequently, $\:\mathrm{e}$ carries no information about expansion or compression, only about shape change. We summarized $\:\mathrm{e}$ into a single per-voxel distortion value using the von Mises equivalent strain $\:{\epsilon\:}_{vm}$, which we defined in direct analogy to the von Mises stress [18, 19]:

$$\:{\epsilon\:}_{vm}=\:\sqrt{\frac{2}{3}\mathrm{e}:\mathrm{e}}$$

Here, $\:\mathrm{e}:\mathrm{e}$ denotes double tensor contraction and the factor $\:\frac{2}{3}$ normalizes $\:{\epsilon\:}_{vm}$ to equal the axial strain under volume-preserving uniaxial deformation; as a result, for example, $\:{\epsilon\:}_{vm}=0.1$ would indicate local shape change comparable to a 10% stretch in one direction at constant volume.

* J* and $\:{\epsilon\:}_{vm}$ were used to generate color-coded heatmaps on 3D brain reconstructions to identify regions of maximum mechanical distortion. Regional values were extracted by overlaying the maps with the segmented brain structures.

### Clinical correlation

Electronic medical records of all included patients were checked for pre- and postoperative clinical evaluations and scores, including Headache Impact Test (HIT-6), which measures the impact of headaches, EuroQol five dimension five-level (EQ-5D-5 L) including the visual analogue scale-based evaluation (EQ-VAS), which measures health-related quality of life, and Montreal Cognitive Assessment (MoCA), to screen for cognitive deficits [20–22]. Clinical scores were correlated with whole-brain von Mises equivalent strain and per-region Jacobian determinant and von Mises equivalent strain in all patients in which perioperative scores were available.

### Statistics

For each brain region, voxel-wise Jacobian determinant and von Mises equivalent strain values were averaged across patients.

For the Jacobian determinant, deviations from unity were assessed using two-tailed one-sample t-tests. As the von Mises equivalent strain is represented by a non-negative number, testing regional means against zero is uninformative. We therefore employed a within-patient normalization approach: for each patient, we calculated the mean and standard deviation of the average strains of all regions. We then calculated the within-patient z-score for each region by subtracting the mean and dividing by the standard deviation (SD) of all regions. To identify regions with significant distortional strain, the resulting z-scores were tested for positive deviation from zero using one-tailed one-sample t-tests. Because within-patient z-scores use a dispersion estimate that extreme regions can inflate, the regional analysis was repeated under robust median/scaled median absolute deviation (MAD) normalization with a one-tailed t-test, with mean/SD normalization and a Wilcoxon signed-rank test, and with median/MAD normalization and a Wilcoxon signed-rank test. The per-region statistical comparisons between SIH group and control group were performed with Welch’s t-test. For the clinical correlation, we calculated the Spearman rank correlation coefficient (r_s_) adjusted for age, sex and the corresponding preoperative baseline score, and reported it with 95% confidence intervals (CI).

To control for multiple comparisons across regions, p-values were adjusted using the Benjamini–Hochberg false discovery rate (FDR) procedure. P-values < 0.05 were considered statistically significant.

## Results

### Patient characteristics

A total of 96 patients with adequate MRIs were included in the deformation and strain analysis. Patients were on average 43.4 ± 11.5 years old, with 62.5% female. Median time between preoperative MRI and surgery was 6 days (interquartile range/IQR: 4–16); median time between surgery and postoperative MRI was 98 days (IQR: 78–123), and median time between pre- and postoperative MRI was 112 days (IQR: 94–147).

### Global deformation patterns of the Jacobian determinant

**Fig. 2:**
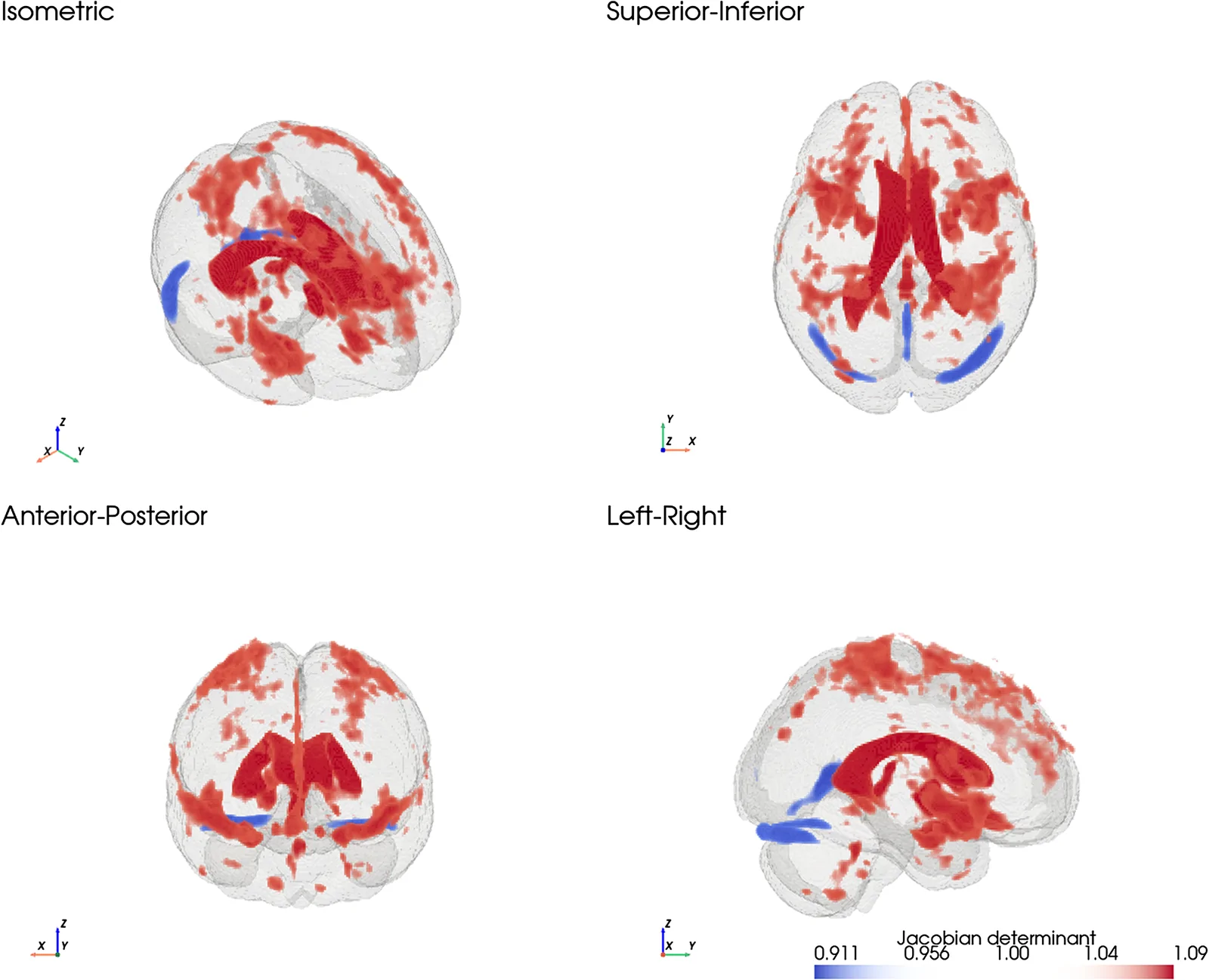
Illustration of the Jacobian determinant from different perspectives. The mode of all included volumetric brain models is represented as a grey, transparent wire mesh. Regions with a Jacobian determinant > 1 (representing expansion) are colored in red; regions with a Jacobian determinant < 1 (representing compression) are colored in blue. For illustrative purposes, only values above the 99th percentile (deviation from unity) are shown, and less extreme values are rendered with increasing transparency

**Fig. 3:**
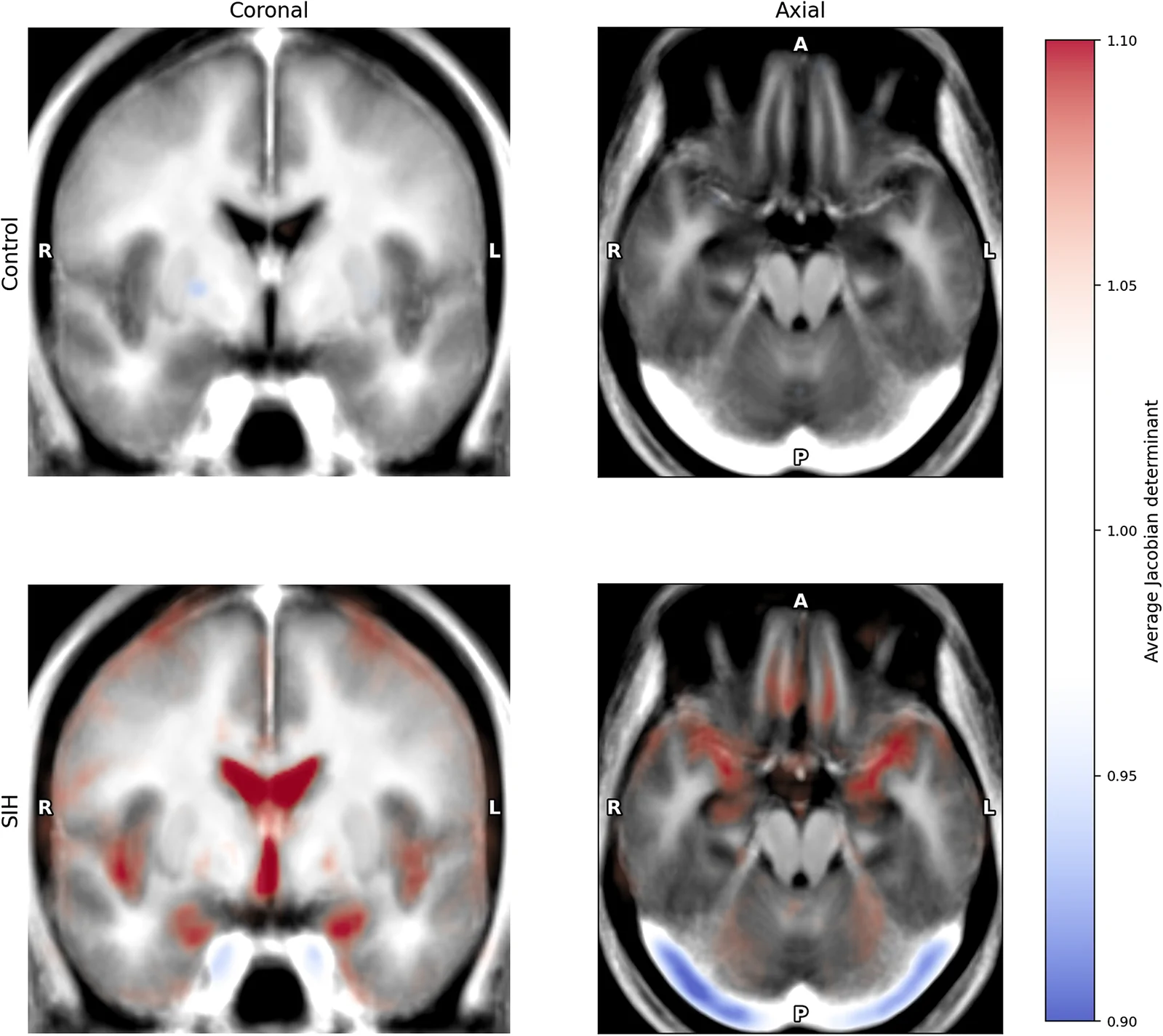
Illustration of the Jacobian determinant in the SIH and control groups in selected representative planes: a coronal view of the average of all included SIH brain models with a section of the lateral and third ventricles, and an axial view at the level of the transverse sinuses. Expanding areas are colored red, compressed areas are colored blue. Orientation is indicated by letters: anterior (A), posterior (P), right (R) and left (L)

The spatial distribution of volumetric changes derived from the Jacobian determinants across patients is illustrated in Fig. 2, with selected representative coronal and axial planes shown and compared to the control group in Fig. 3. The most pronounced expansion was seen around the lateral ventricles and to a lesser degree in apical and lateral frontobasal cortical regions. Conversely, the most prominent compression was found along the transverse and straight venous sinuses. Compared to the SIH group, the control group showed no relevant tissue expansion or contraction.

### Distribution of distortional strain

**Fig. 4:**
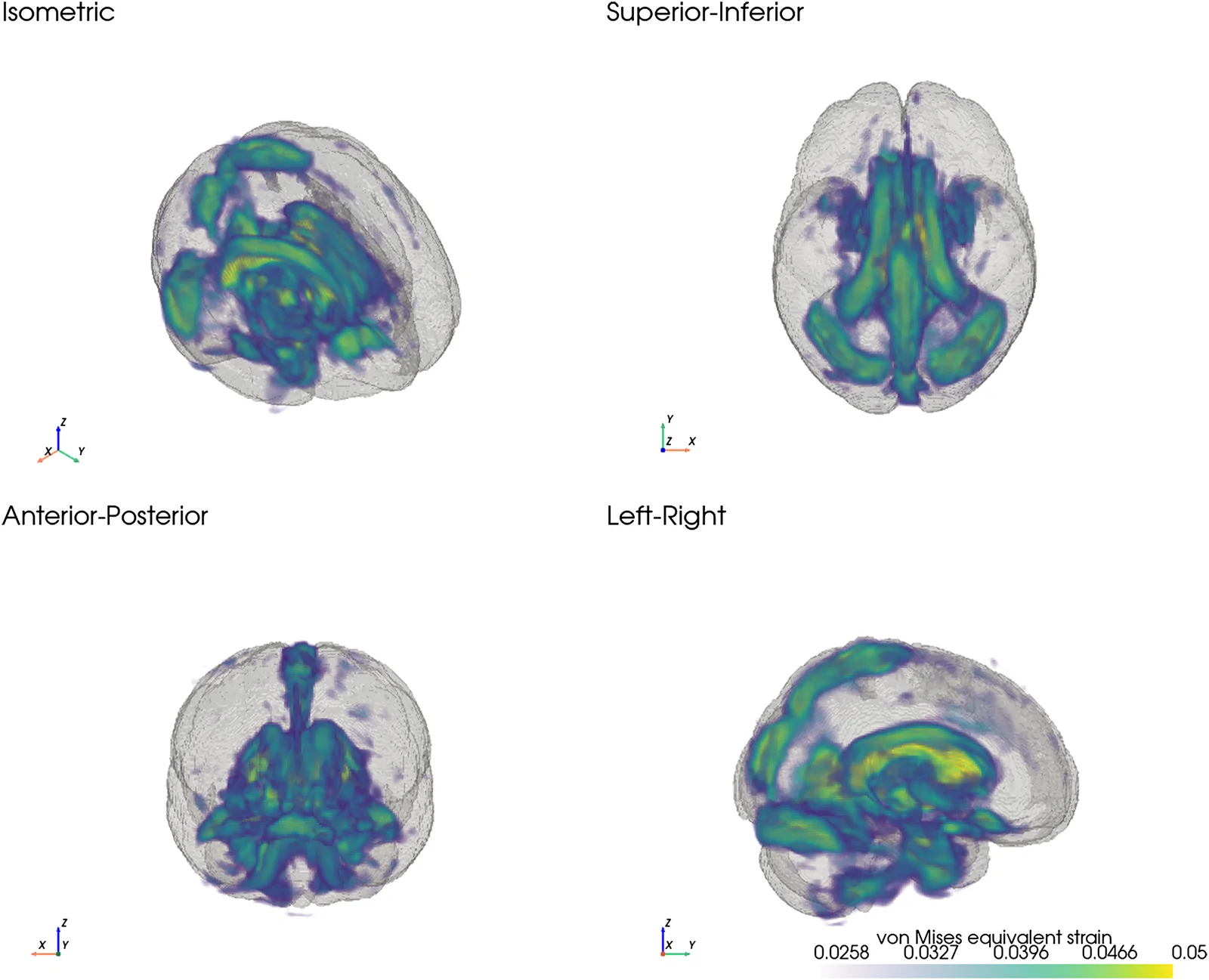
Illustration of the von Mises equivalent strain from different perspectives. The mode of all included volumetric brain models is represented as a grey, transparent wire mesh. Regions with relevant strain are colored with intensity based on the *viridis* color scale. For illustrative purposes, only values above the 95th percentile (deviation from zero) are shown, and lower values are rendered with increasing transparency

**Fig. 5:**
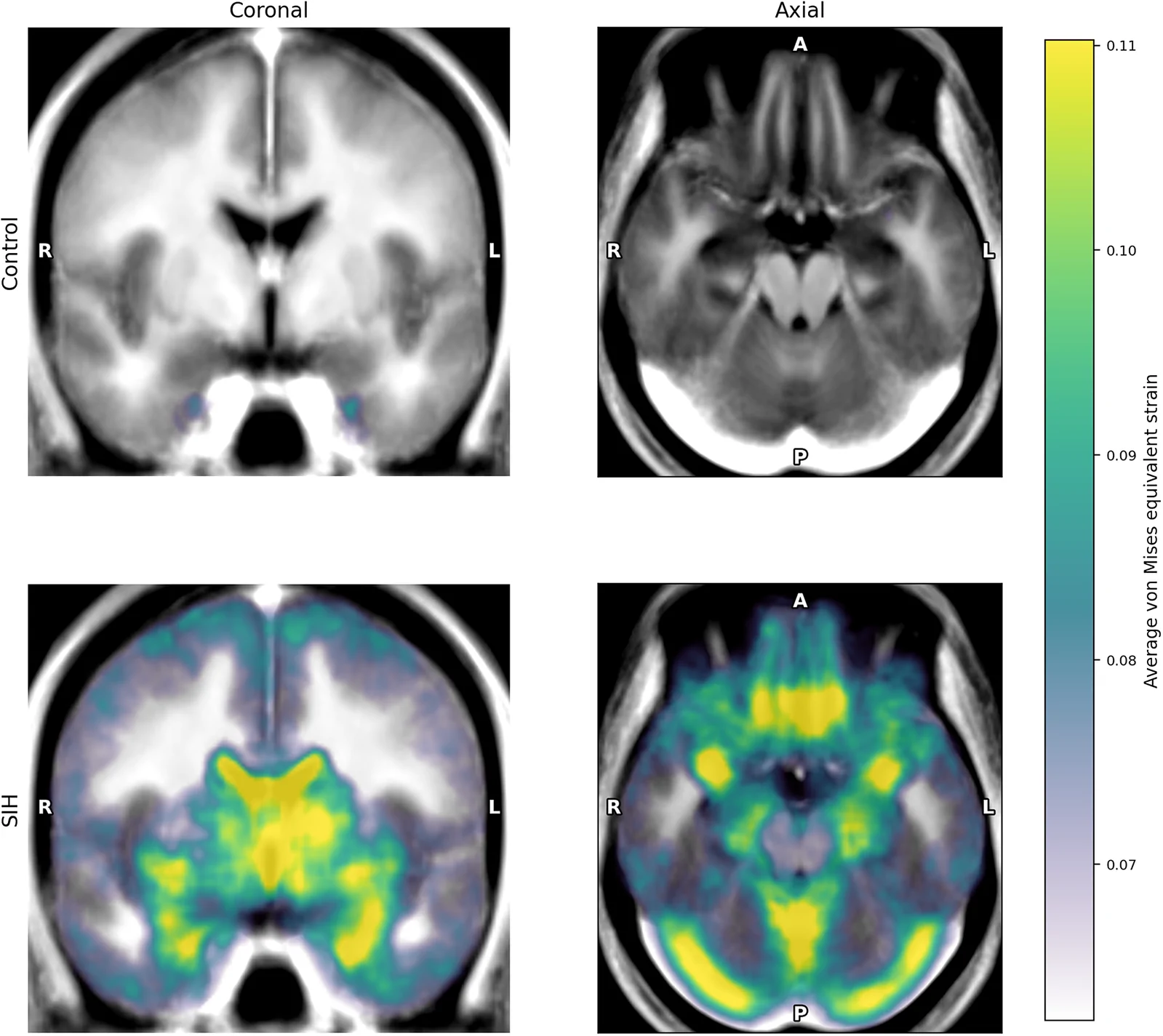
Illustration of the von Mises equivalent strain in the SIH and control groups in selected representative planes: a coronal view of the average of all included SIH brain models with a section of the lateral and third ventricles, and an axial view at the level of the transverse sinuses. Regions with relevant strain are colored with intensity based on the *viridis* color scale. Orientation is indicated by letters: anterior (A), posterior (P), right (R) and left (L)

A visual representation of the average distortional strain and its spatial distribution is shown in Fig. 4, with selected representative coronal and axial planes shown and compared to the control group in Fig. 5. The highest distortion was observed in the ventricular regions, mostly along the lateral ventricular walls. Here, high distortional strain coincided with pronounced volumetric expansion. This co-occurrence is mechanically expected at a fluid-filled cavity where the deformation field at the ventricular margin necessarily combines volume change with shape change. Relevant distortion was also observed along the transverse and superior sagittal sinuses, where it likewise accompanies the volumetric compression described above. Distortional strain was further increased in the frontobasal and temporomesial brain regions, in particular in the ventromedial prefrontal cortex, orbitofrontal cortex, temporal poles, hippocampal and parahippocampal regions, and the amygdala. In contrast to the ventricles and venous sinuses, these regions exhibited substantial distortional strain despite only moderate volumetric changes (Jacobian determinant = 1.021–1.067), indicating that mechanical deformation of the brain parenchyma is not limited to areas of maximal volumetric expansion. Compared to the SIH group, the control group showed no relevant distortional strain.

### Regional patterns of volumetric change and mechanical strain

Regional averages of the Jacobian determinant and distortional strain for major brain regions are summarized in Tables 1 and 2 respectively, with the complete set of segmented regions provided in Supplement 1 and Supplement 2, respectively. Consistent with the global maps, most regions demonstrated statistically significant volumetric expansion following surgical leak closure. The largest Jacobian determinant changes were observed in ventricular regions (right lateral ventricle: 1.174, left lateral ventricle: 1.176), with smaller changes toward the cortical surface. Distortional strain was very high around the lateral (right: 0.127, left: 0.130) and third (0.126) ventricles; on average, none of the major lobes showed significantly elevated strain. In direct comparison with the control group, except for the brainstem, all regions showed statistically significant expansion or compression.

**Table 1:** Overview of results for the Jacobian determinant for all major brain regions, with means and standard deviations (SD)

Region	* J*- Mean	* J*- SD	*P*-Value	Significant
Brainstem	1.001	0.037	0.696	No
Right cerebellum	1.026	0.060	5.86 * 10^− 5^	**Yes**
Left cerebellum	1.021	0.053	2.24 * 10^− 4^	**Yes**
Right frontal lobe	1.035	0.050	8.15 * 10^− 10^	**Yes**
Left frontal lobe	1.032	0.049	7.42 * 10^− 9^	**Yes**
Right temporal lobe	1.024	0.040	5.09 * 10^− 8^	**Yes**
Left temporal lobe	1.026	0.042	1.62 * 10^− 8^	**Yes**
Right parietal lobe	1.041	0.053	2.75 * 10^− 11^	**Yes**
Left parietal lobe	1.037	0.052	2.96 * 10^− 10^	**Yes**
Right occipital lobe	1.026	0.045	1.57 * 10^− 7^	**Yes**
Left occipital lobe	1.025	0.044	1.96 * 10^− 7^	**Yes**
3rd ventricle	1.120	0.164	1.57 * 10^− 10^	**Yes**
4th ventricle	1.070	0.121	1.81 * 10^− 7^	**Yes**
Right lateral ventricle	1.174	0.276	1.75 * 10^− 8^	**Yes**
Left lateral ventricle	1.176	0.262	2.35 * 10^− 9^	**Yes**

*P*-values are unadjusted; significance is determined after FDR correction

**Table 2:** Overview of results for the von Mises equivalent strain for all major brain regions, with means and standard deviations (SD)

Region	$\:{\epsilon\:}_{vm}$- Mean	$\:{\epsilon\:}_{vm}$- SD	*P*-Value	Significant
Brainstem	0.069	0.031	> 0.999	No
Right cerebellum	0.078	0.033	0.992	No
Left cerebellum	0.076	0.032	> 0.999	No
Right frontal lobe	0.074	0.027	> 0.999	No
Left frontal lobe	0.074	0.027	> 0.999	No
Right temporal lobe	0.072	0.023	> 0.999	No
Left temporal lobe	0.072	0.024	> 0.999	No
Right parietal lobe	0.071	0.028	> 0.999	No
Left parietal lobe	0.070	0.027	> 0.999	No
Right occipital lobe	0.078	0.030	0.989	No
Left occipital lobe	0.077	0.029	0.999	No
3rd ventricle	0.126	0.085	4.39 * 10^− 14^	**Yes**
4th ventricle	0.081	0.038	0.818	No
Right lateral ventricle	0.127	0.102	4.91 * 10^− 9^	**Yes**
Left lateral ventricle	0.130	0.100	1.41 * 10^− 10^	**Yes**

*P*-values are unadjusted; significance is determined after FDR correction

**Fig. 6:**
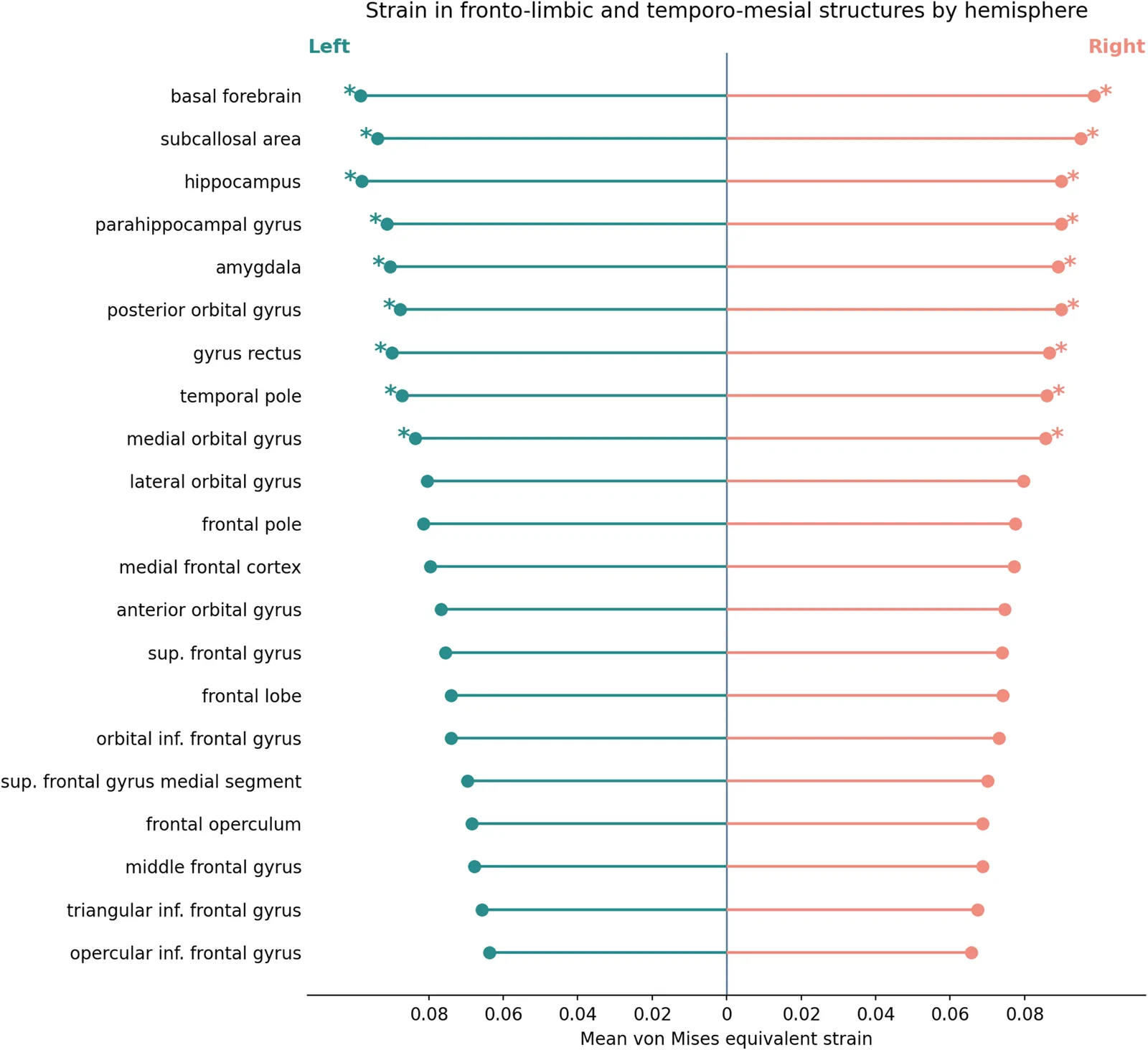
Bar plot showing the von Mises equivalent strain for fronto-limbic and temporo-mesial structures on the left and right. Asterisks indicate regions with significantly elevated strain after correction for multiple testing

Significantly elevated distortional strain was consistently observed across fronto-limbic and temporo-mesial structures (Fig. 6). These included the subcallosal area (mean strain = 0.095/ 0.094 on the right/ left), basal forebrain (0.099/ 0.098), hippocampus (0.090/ 0.098), parahippocampal region (0.090/ 0.091), amygdala (0.089/ 0.090), and temporal pole (0.086/ 0.087) (all *p* < 0.001). Similar values were also observed in the ventromedial prefrontal cortex and orbitofrontal cortex (0.073–0.090). All regions significant in the primary analysis remained significant under robust normalization, and compared to the healthy control group, all regions showed elevated strain; additional statistical testing is detailed in Supplement 2.

### Clinical correlation

**Fig. 7:**
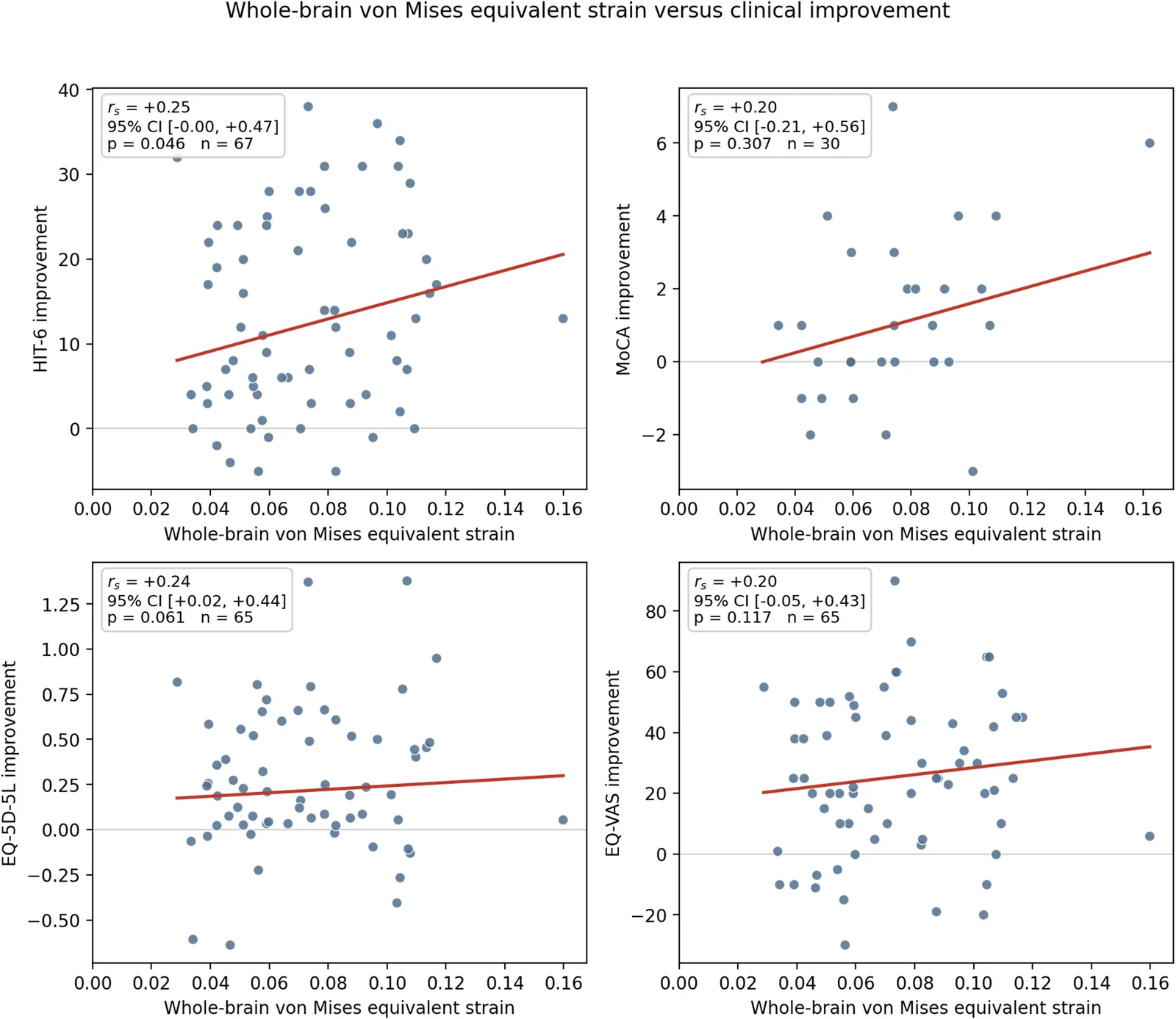
Whole-brain von Mises equivalent strain across the perioperative interval versus clinical improvement. Annotated coefficients (r_s_) are partial Spearman rank correlations adjusted for age, sex and the corresponding baseline score, with 95% confidence intervals. Regression lines are unadjusted Theil–Sen fits shown for visualisation only

Clinical correlation between whole-brain von Mises equivalent strain and clinical scores are shown in Fig. 7; a summary of clinical score distribution and detailed per-region correlation analysis can be found in Supplement 3. We found a modest association between whole-brain strain and improvement in HIT-6 scores (r_s_ =0.25; CI: 0.00–0.47; *p* = 0.046); other clinical scores showed comparable effect sizes, but with markedly wider confidence intervals for the MoCA score correlation. In the per-region analysis, after FDR correction, none of the brain regions showed a statistically significant correlation with any clinical score. The highest correlation between strain and perioperative MoCA improvement was found in the left temporal pole (r_s_ =0.40; CI: 0.12–0.65; *p* = 0.037), but the correlation was not significant after correction for multiple testing.

## Discussion

This study provides a quantitative assessment of brain tissue deformation in spontaneous intracranial hypotension (SIH). We demonstrate that SIH is not only associated with “brain sagging” and measurable volumetric changes, but also with significant mechanical strain. Hot spots with elevated mechanical strain turned out to be in the frontobasal and temporomesial brain regions, independent of volumetric redistribution. Whether these intriguing findings offer a mechanistic explanation for the clinical manifestations of patients with SIH needs further study.

The strain analysis revealed a spatial organization with pronounced strain in the ventromedial prefrontal cortex and mesial temporal lobes. When visualized across the brain, these regions form a spatially coherent pattern extending from the temporomesial structures toward the ventromedial prefrontal and orbitofrontal cortices. This distribution corresponds to the anatomical organization of fronto-limbic networks that connect the hippocampus, amygdala, basal forebrain, and medial prefrontal cortex [23–25].

These structures constitute the core of fronto-limbic circuits supporting behavioral and executive control, emotional regulation, cognition, memory, and affective processing. They are central to the pathophysiology of bvFTD and related syndromes [26–28]. Previous reports of “brain sagging dementia” or “spinal dementia” have been descriptive, highlighting symptomatic similarities and radiological signs of downward brain displacement. Our results extend these observations by providing quantitative data hinting that mechanical stress and SIH-related symptoms like cognitive and behavioral changes may relate to region-specific mechanical distortion within cognition-relevant networks. Emerging hypotheses, e.g., that chronic, region-specific strain may contribute to potential axonal injury, synaptic dysfunction, and possible structural neurodegeneration, require further investigation. The prompt improvement in some patients after closure of a spinal leak would favor the view that the observed deformation pattern constitutes a mechanically induced network dysfunction rather than irreversible tissue loss. Distortional strain may transiently alter the efficiency of connectivity within fronto-limbic networks and is consistent with the reversibility of cognitive symptoms after leak closure [3]. On the other hand, it might be worthwhile to analyze whether residual symptoms could be related to more permanent mechanically induced tissue dysfunction or damage.

Similar concepts have been proposed in other neurological disorders where mechanical forces play a role, such as traumatic brain injury and chronic traumatic encephalopathy, supporting the plausibility of a strain-mediated pathogenic mechanism [29–31]. 

Our analysis based on the Jacobian determinant demonstrated structured expansion and compression patterns following surgical leak closure (e.g., ependymal and parenchymal re-expansion adjacent to the ventricles and compression of brain parenchyma along venous sinuses). Analogous volumetric changes have already been demonstrated by Zander et al. and Mehan et al. using manual measurements and align with the re-expansion of CSF spaces and the normalization of venous engorgement seen on brain MRIs [32, 33]. 

Our Jacobian determinant maps extend these observations from compartment-level volume measurements to a voxel-wise spatial distribution, showing where within the brain expansion and compression occur. A comparable regional distinction has been described using magnetic resonance elastography in normal-pressure hydrocephalus, where periventricular stiffness changes partly normalize after shunting [34, 35], indicating that tissue immediately around the ventricles responds to volume change differently from the rest of the parenchyma — the same regional contrast we observed in our strain maps.

Functional imaging studies point in the same direction. Perfusion imaging in SIH is limited to case observations, in which SPECT showed reduced cerebral perfusion in the sagging brain that improved after epidural patching, in parallel with cognitive recovery [36]. Functional MRI is more informative: imaging before and one month after leak closure, Amemiya et al. found abnormal activity in the bilateral orbitofrontal and medial frontal cortices with altered default-mode network activity, normalizing as working memory recovered [37]. This independently derived abnormality involves the same orbitofrontal and ventromedial prefrontal territory in which we measured disproportionate strain, suggesting a mechanical substrate for a reversible functional deficit. Multimodal imaging in frontotemporal brain sagging syndrome likewise implicates altered frontal connectivity rather than atrophy [38].

Although direct comparisons are limited, earlier imaging studies of SIH have emphasized global brain sagging and secondary signs such as venous engorgement, subdural collections, and reduced cisternal spaces – all visible with the naked eye. Dobrocky et al. introduced an MRI-based SIH score that captured these features but did not quantify local deformation [10]. Our results may provide a missing link by showing that deformation is not uniformly distributed but concentrated in regions implicated in higher cognitive and behavioral control.

Concerning the correlation with clinical symptoms, we found a modest association between perioperative headache improvement as measured by the HIT-6 score and increased whole-brain von Mises equivalent strain. The correlation is only weak, and is confounded by postoperative rebound hypertension and residual symptoms, as was described in previous publications [39, 40]. Comparing perioperative whole-brain strain with improvements in quality-of-life scores such as EQ-5D-5 L and cognitive assessment tools such as MoCA showed positive correlations that were similar in magnitude to HIT-6, but with markedly wider confidence intervals, especially for MoCA. The cognitive analysis should be regarded as underpowered rather than negative: Out of the 96 subjects included in this study, only 30 had both pre- and post-operative MoCA scores. In addition, only a minority of subjects showed cognitive impairment, with a median preoperative MoCA score of 28. The low proportion of cognitively impaired patients in our population is aggravated by our study design: Exclusion of patients with subdural hygroma or hematoma, whose effusions generate deformation far exceeding that of sagging alone, further removes the subgroup with the most severe symptoms and the largest shifts. Establishing or refuting a link between strain and cognition will require a larger cohort with protocol-driven neurocognitive testing.

Several other limitations should be acknowledged. First, the retrospective, single-center design may introduce selection bias, and the study was restricted to patients with sufficient pre- and postoperative imaging, potentially introducing bias. Second, although diffeomorphic registration and AI-based segmentation provide robust tools for quantifying deformation, registration errors and segmentation inaccuracies cannot be fully excluded, despite verification by the authors. The healthy control group provides an empirical estimate of this residual noise floor: applying the identical pipeline to subjects without brain sagging yielded almost no measurable expansion, compression or distortional strain, whereas the deformation measured in patients was severalfold larger. The control cohort was, however, younger than our patients (19–23 years versus 43.4 ± 11.5 years) and was imaged without contrast agent on different scanners. These differences would, if anything, be expected to increase rather than decrease apparent deformation in the control group, so we regard the comparison as conservative, but it is not perfectly matched. Third, FDR correction assumes independence among tests; neighbouring regions are spatially and physiologically positively correlated, so the effective number of independent tests is smaller than the nominal one, which renders our regional testing conservative. Finally, the observed association between mechanical strain and symptomatic improvement is correlational; causal inference is not possible from these data, and prospective studies with systematic clinical and especially neurocognitive testing will be required.

## Conclusions

Our model shows that spinal leaks in patients with spontaneous intracranial hypotension (SIH) are associated with quantifiable region-specific mechanical strain on the brain. The overlap between deformation hotspots and fronto-limbic and temporomesial regions may provide an intriguing explanation for the clinical phenotype observed in some patients.

## Supplementary Information

Below is the link to the electronic supplementary material.


Supplementary Material 1



Supplementary Material 2



Supplementary Material 3


## Data Availability

Parts of the dataset containing data from patients who agreed to share it with other researchers will be made available upon reasonable request to the corresponding author.

## Abbreviations

AI: Artificial intelligence
bvFTD: Behavioral variant frontotemporal dementia
CSF: Cerebrospinal fluid
FDR: False discovery rate
MRI: Magnetic resonance imaging
SIH: Spontaneous intracranial hypotension
